# Outcome analysis and risk factors for postoperative colonic ischaemia after aortic surgery

**DOI:** 10.1007/s00423-020-01964-2

**Published:** 2020-08-21

**Authors:** Dmitriy I. Dovzhanskiy, Moritz S. Bischoff, Christopher D. Wilichowski, Fabian Rengier, Anna Klempka, Dittmar Böckler

**Affiliations:** 1 Department of Vascular and Endovascular Surgery, University Hospital Heidelberg, Im Neuenheimer Feld 110, 69120 Heidelberg, Germany; 2 Clinic for Diagnostic and Interventional Radiology, University Hospital Heidelberg, Im Neuenheimer Feld 110, 69120 Heidelberg, Germany

**Keywords:** Colonic ischaemia, Aortic surgery, Abdominal aortic aneurysm, Ischaemic colitis, Outcome, Risk factors, Vascular surgery, Matched-pair analysis

## Abstract

**Purpose:**

Colonic ischaemia (CI) represents a serious complication after aortic surgery. This study aimed to analyse risk factors and outcome of patients suffering from postoperative CI.

**Methods:**

Data of 1404 patients who underwent aortic surgery were retrospectively analysed regarding CI occurrence. Co-morbidities, procedural parameters, colon blood supply, procedure-related morbidity and mortality as well as survival during follow-up (FU) were compared with patients without CI using matched-pair analysis (1:3).

**Results:**

Thirty-five patients (2.4%) with CI were identified. Cardiovascular, pulmonary and renal comorbidity were more common in CI patients. Operation time was longer (283 ± 22 vs. 188 ± 7 min, *p* < 0.0001) and blood loss was higher (2174 ± 396 vs. 1319 ± 108 ml, *p* = 0.0049) in the CI group. Patients with ruptured abdominal aortic aneurysm (AAA) showed a higher rate of CI compared to patients with intact AAA (5.4 vs. 1.9%, *p* = 0.0177). CI was predominantly diagnosed by endoscopy (26/35), generally within the first 4 postoperative days (20/35). Twenty-eight patients underwent surgery, all finalised with stoma creation. Postoperative bilateral occlusion and/or relevant stenosis of hypogastric arteries were more frequent in CI patients (57.8 vs. 20.8%, *p* = 0.0273). In-hospital mortality was increased in the CI group (26.7 vs. 2.9%, *p* < 0.0001). Survival was significantly reduced in CI patients (median: 28.2 months vs. 104.1 months, *p* < 0.0001).

**Conclusion:**

CI after aortic surgery is associated with considerable perioperative sequelae and reduced survival. Especially in patients at risk, such as those with rAAA, complicated intraoperative course, severe cardiovascular morbidity and/or perioperative deterioration of the hypogastric perfusion, vigilant postoperative multimodal monitoring is required in order to initiate diagnosis and treatment.

**Electronic supplementary material:**

The online version of this article (10.1007/s00423-020-01964-2) contains supplementary material, which is available to authorized users.

## Introduction

Aortic pathologies, including abdominal aortic aneurysm (AAA), belong to the most complex part of modern vascular surgery, requiring ongoing development of technological and treatment strategies. Although aortic surgery can be safely performed in high-volume centres [1], certain severe complications remain common for these patients. Colonic ischaemia (CI) is one of the most serious postoperative adverse events with high in-hospital mortality. The majority of publications addressing CI during the last decade have focussed on the pathogenesis or early diagnosis of CI, whereas its impact on the surgical short- and long-term outcomes has been less well described [2–4]. A recent analysis of insurance data revealed a worsening of in-hospital outcomes and long-term survival in patients with CI, where endovascular techniques (EVAR) seemed to be protective after repairs of both ruptured and intact AAA [5]. Williamson et al. [4] also reported a reduced incidence of CI after EVAR in their meta-analysis compared to open repair (OR). At the same time, the medical and surgical features of the postoperative period and changes in colonic blood supply in patients with and without CI are less well-known. This study aimed to analyse the clinical course of CI after aortic surgery, and to describe the short- and long-term results for these patients compared with patients without CI.

## Material and methods

### Patients

For this study, the medical case files of 1404 patients who underwent aortic surgery between 2001 and 2012 in the Department of Vascular and Endovascular Surgery, University of Heidelberg, Germany, were retrospectively analysed with respect to postoperative CI occurrence. CI was diagnosed either by postoperative endoscopy or exploratory laparotomy. Clinical data were obtained from the institution’s database and patients’ medical records. The study was approved by the Ethics Committee of the Medical Faculty of Heidelberg (protocol number: S-110/2012).

### Data acquisition and follow-up

Data were extracted using demographic parameters (age, sex), main diagnosis and type of operative procedure. The features of the clinical presentation of CI were described in a descriptive fashion. The co-morbidities, preoperative medication and intraoperative parameters were analysed to identify CI risk factors. Moreover, intraoperative parameters (operating time, intraoperative blood loss, transfusion of red blood cells (RBCs) and fresh frozen plasma (FFP) and intraoperative hypotension or hypothermia) were evaluated.

Preoperative as well as postoperative computed tomography angiography (CTA) scans were evaluated by an experienced radiologist regarding the patency rate (occlusion and/or stenosis ≥ 70%) of the inferior mesenteric artery and bilateral hypogastric perfusion.

Analysis of postoperative morbidity and mortality included postoperative respiratory and cardiovascular complications; secondary postoperative bleeding, blood transfusion within the first three postoperative days; frequency and infusion rate of postoperative vasopressor therapy; and incidence of acute kidney injury (AKI). The definition of AKI was based on the acute kidney injury network (AKIN) criteria [6]. Additionally, the lengths of the intensive care unit (ICU) and in-hospital stays were evaluated. For follow-up (FU), the respective registration offices were contacted in 2015 and asked for reporting information on mortality and/or survival data for all patients.

### Matched-pair analysis

To compare the outcome of surgical management and survival in patients with and without CI, we performed a case-control study with a matched design. The following parameters were used to match one CI patient (case) to three patients without CI (controls): age (± 5 years) and sex of the patients and type of operation and primary diagnosis. Matching controls were identified from the same database as the CI patients.

### General management of CI after aortic surgery

At the authors’ institution, the diagnostic gold standard for suspected CI after aortic surgery is endoscopy. Of note, endoscopy after aortic surgery is not undertaken on a routine basis. The technique is generally performed to at least 40 cm in all patients. The rectosigmoid junction is thereby always examined. Based on clinical and endoscopic findings (i.e. superficial mucosal vs. transmural ischaemia), the patient is treated by conservative or surgical means. Conservative therapy usually comprises bowel rest, intravenous hydration, parenteral nutrition and broad-spectrum antibiotic therapy. Along with meticulous clinical monitoring, repeat colonoscopy is performed at regular intervals, to ascertain the response to management. In case of progression, the patient is treated with exploratory laparotomy and resection of the ischaemic bowel tissue. Patients in whom transmural ischaemia is detected undergo immediate exploratory laparotomy and resection of the ischaemic bowel tissue, predominantly colonic resection under avoidance of a primary anastomosis.

### Statistical analysis

GraphPad Prism version 5 for Windows (GraphPad Software, Inc., San Diego, CA, USA) was used for statistical analysis. Quantitative variables are expressed as either the median with range or the mean with standard deviation. Comparisons between subgroups of patients with respect to quantitative variables were performed using the Mann–Whitney *U* test or the Kruskal–Wallis test. Categorical variables were analysed using Fisher’s exact test. Overall survival was defined as the time from the date of the surgery to either death from any cause or the last FU. Survival estimates were calculated using Kaplan–Meier analysis. Differences between survival curves were examined with the log-rank test. Two-sided *p* values were always computed, and a difference was considered statistically significant at *p* < 0.05.

## Results

A total of 1404 patients underwent aortic surgery for aneurysms between January 2001 and December 2012 in the Department of Vascular Surgery, University of Heidelberg, Germany. Thirty-five of these patients were identified with CI, representing 2.4% of the sample. Patients’ characteristics are summarised in Table 1. The median age of the patients with CI was 70.8 years (range, 52–83 years). CI was more frequently found in men, with a female-to-male ratio of 3:32. The diagnoses leading to aortic surgery were intact AAA (*n* = 25), ruptured AAA (*n* = 8) and proximal anastomotic aneurysm after previous OR (*n* = 2). Regarding the influence of aneurysm rupture on CI, there was a significant increase in the incidence of CI in patients with rAAA (5.4 vs. 1.9%, *p* = 0.0177). CI occurred both after OR (2.7%) and EVAR (2.1%). Statistical analysis revealed no significant differences between the two types of repair (*p* = 0.5897, Table 2).

**Table 1:** Demographic and clinical data of patients with colonic ischaemia after aortic surgery

Total number of patients with CI	35
Male, *n* (%)	3 (94.3%)
Age, years (range)	70.8 ± 1.1 (52–83)
Main diagnosis, *n* (%)	
AAA	25 (71.4%)
rAAA	8 (22.9%)
Proximal anastomotic aneurysm after previous aortic surgery	2 (5.7%)

*CI*, colonic ischaemia; *AAA*, abdominal aortic aneurysm; *rAAA*, ruptured abdominal aortic aneurysm

**Table 2:** Occurrence of colonic ischaemia after aortic surgery

	CI	Operations 2001–2012	*P*
OR	25 (2.7%)	918	0.5897(OR vs. EVAR)
EVAR	10 (2.1%)	486	
rAAA	8 (5.4%)	148	0.0177 (rAAA vs. iAAA)
iAAA	25 (1.9%)	1256	
Total	*35 (2.4%)*	*1404*	

*CI*, colonic ischaemia; *OR*, open repair; *EVAR*, endovascular aortic aneurysm repair; *AAA*, abdominal aortic aneurysm; *rAAA*, ruptured abdominal aortic aneurysm

The clinical symptoms of CI after aortic surgery are presented in Table 3. CI was diagnosed in 25.7% (9 patients) during the first 2 days and in 31.4% (11 patients) during days 3–4 after surgery. Abdominal symptoms existed in 27 (77.1%) and diarrhoea in 13 (37.1%) patients. Worsening of the general condition as the first symptom was recorded in 17 (48.6%) patients. In most cases, CI was confirmed by colonoscopy (*n* = 26, 74.2%). In nine (25.8%) patients, the diagnosis was made by explorative re-laparotomy. Elevation of serum lactate was registered in only 11 (31.4%) cases. Most patients had an increase of C-reactive protein (CRP), with a mean of 224.0 ± 18.8 mg/l. One-fifth of patients (*n* = 7, 20%) could be treated conservatively, but 31 patients (80%) required surgical treatment. The intraoperative status showed ischaemia localised in the left hemicolon in 20 (71.4%) cases and in the right hemicolon in one (3.6%) case; in seven (25%) cases, there was a total colon lesion. The perforation was verified in five (17.6%) patients. Fourteen (50%) patients received a subtotal colectomy, while 13 patients had left hemicolectomy or resection of colon sigmoideum and one patient received right hemicolectomy. Surgery was finalised with stoma creation in all cases. Seventeen patients (60.7%) had wound healing problems, and 11 (39.3%) patients underwent secondary surgery. The in-hospital mortality rate was 31.4% (*n* = 11): most patients (*n* = 8, 72.7%) died due to sepsis, two (18.2%) patients died due to respiratory or cardiac failure, and one died due to intracerebral bleeding. Of 24 (68.6%) patients who were alive, 10 (41.6%) were discharged home, 10 (41.6%) were transferred to another hospital, and four (16.8%) were transferred to a rehabilitation institution. During FU, eight (28.5%) patients underwent stoma closure, and 12 (34.3%) patients died after discharge. Of these, six patients died due to cardiac reasons, one due to cachexia, one due to pneumonia and one due to metastatic lung cancer. The cause of death of the remaining three patients remains unclear.

**Table 3:** Clinical presentation of colonic ischaemia after aortic surgery

Time point of diagnosis of colonic ischemia	*n* = 35
1–2 days	9 (25.7%)
3–4 days	11 (31.4%)
5–9 days	5 (14.3%)
> 10 days	10 (28.6%)
Clinical symptomatic	
Abdominal symptoms	27 (77.1%)
Diarrhoea	13 (37.1%)
Worsening of general condition	17 (48.6%)
Diagnostics	
Colonoscopy	26 (74.2%)
Explorative laparotomy	9 (25.7%)
Serum lactate > 20 mg/dl	11 (31.4%)
CRP (mg/l)	224.0 ± 18.8
Treatment	
Conservative treatment	7 (20%)
Surgical treatment	28 (80%)
Right hemicolectomy	1 (3.6%)
Left hemicolectomy	6 (21.4%)
Subtotal colectomy	14 (50%)
Resection of colon sigmoideum	7 (25%)
Stoma creation	28 (100%)
Intraoperative status (localisation/perforation)	
Left hemicolon	20 (71.4%)
Right hemicolon	1 (3.6%)
Total colon	7 (25%)
Perforation	5 (17.6%)
In-hospital mortality	11 (31.4%)
Sepsis	8 (72.7%)
Respiratory or cardiac failure	2 (18.2%)
Intracranial bleeding	1 (9.1%)
Discharge alive	24 (68.6%)
At home	10 (41.6%)
External hospital	10 (41.6%)
Rehabilitation institution	4 (16.8%)

The matched-pair data on preoperative risk factors of CI (co-morbidities and preoperative medication) are shown in Table 4. Both matched-pair groups were comparable in height, weight and body mass index (BMI). Compared with patients without CI, there were significant differences in cardiac co-morbidities. The rate of coronary artery disease (65.7 vs. 44.2%, *p* = 0.0326) and previous myocardial ischaemia (51.4 vs. 24%, *p* = 0.0053) were increased in CI patients. The rate of chronic heart failure (60 vs. 26.9%, *p* = 0.0009) was also more than twofold higher in the CI group. Peripheral artery disease (37.1 vs. 14.4%, *p* = 0.0066), chronic obstructive pulmonary disease (42.9 vs. 18.3%, *p* = 0.0058) and anamnestic nicotine abuse (77.1 vs. 45.2%, *p* = 0.0015) were significantly more frequent in the CI group. Additionally, CI was associated with a higher incidence of preoperative renal dysfunction: 48.6% of CI patients had elevated (> 1.2 mg/dl) serum creatinine levels, compared with only 26.9% of patients without CI (*p* = 0.0224).

**Table 4:** Analysis of co-morbidities of matched patients with/without colonic ischaemia

	CI (*n* = 35)	Control (*n* = 104)	*P*	Odds ratio
Coronary artery disease	23 (65.7%)	46 (44.2%)	*0.0326*	*2.42 (1.09–5.37)*
Previous myocardial infarction	18 (51.4%)	25 (24%)	*0.0053*	*3.35 (1.50–7.46)*
Chronic heart failure	21 (60%)	28 (26.9%)	*0.0009*	*4.07 (1.82–9.09)*
Peripheral artery disease	13 (37.1%)	15 (14.4%)	*0.0066*	*3.51 (1.46–8.43)*
Chronic obstructive pulmonary disease	15 (42.9%)	19 (18.3%)	*0.0058*	*3.36 (1.46–7.73)*
Nicotine abuse	27 (77.1%)	47 (45.2%)	*0.0015*	*4.09 (1.70–9.85)*
Preoperative elevated creatinine (> 1.2 mg/dl)	17 (48.6%)	28 (26.9%)	*0.0224*	*0.02 (1.16–5.66)*
Previous abdominal surgery	9 (25.7%)	20 (19.2%)	1.0000	1.05 (0.44*–*2.53)
Diabetes mellitus	9 (25.7%)	13 (12.5%)	0.1048	2.42 (0.93*–*6.30)

Italicized entries mean statistically significant results

*CI*, colonic ischaemia

Comparing intraoperative parameters (Suppl. Table 1), operation time was significantly longer (283 ± 22 vs. 188 ± 7 min, *p* < 0.0001) and blood loss was significantly higher (2174 ± 396 vs. 1319 ± 108 ml, *p* = 0.0049) in the CI group. Moreover, the number of patients with a blood loss ≥ 2000 ml (42.9 vs. 15.4%, *p* = 0.0017), intraoperative hypothermia (60 vs. 32.7%, *p* = 0.0053) and hypotension (40 vs. 20.2%, *p* = 0.0251) was elevated in the CI group. The patients with CI received intraoperative transfusion of RBCs more often and in higher volumes (frequency, 57.1 vs. 30.8%; *p* = 0.0082; volume, 4.7 ± 1.3 vs. 1.6 ± 0.4 units; *p* = 0.0021) than control patients. The same was true for FFP transfusion (frequency, 57.1 vs. 30.8%; *p* = 0.0082; volume, 4.0 ± 0.9 vs. 1.4 ± 0.3 units; *p* = 0.0011; Suppl. Table 1).

The analysis of pre- and postoperative colon blood supply in patients with and without CI is shown in Table 5. Preoperative CTA scans were available for 22 patients and postoperative CTA scans for 23 patients with CI. In the control group, a preoperative scan was available for 75 patients and a postoperative CTA for 48 patients. There were no differences in the rates of preoperative occlusion of inferior mesenteric artery and stenosis or occlusions of hypogastric arteries. In contrast, the rate of postoperative lesions (> 70% stenosis or occlusion) of both hypogastric arteries was significantly higher in the CI group (57.8 vs. 20.8%, *p* = 0.0273).

**Table 5:** Blood supply of colon in patients with/without colonic ischaemia

	CI	Control	*P*	Odds ratio
Preoperative occlusion of IMA	9 (40.1%, *n* = 22)	34 (45.3%, *n* = 75)	0.8094	0.83 (0.32*–*2.19)
Preoperative lesion of both IIA	10 (45.5%, *n* = 22)	19 (25.3%, *n* = 75)	0.1101	2.46 (0.91*–*6.60)
Preoperative occlusion of at least one IIA	3 (13.6%, *n* = 22)	7 (9.3%, *n* = 75)	0.6901	1.53 (0.36*–*6.51)
Postoperative lesion of both IIA	11 (57.8%, *n* = 23)	10 (20.8%, *n* = 48)	*0.0273*	*3,48 (1.19–10.20)*

CI, colonic ischaemia; *IMA*, inferior mesenteric artery; *IIA*, internal iliac artery

Comparing postoperative morbidity, prolonged mechanical ventilation over 48 h (40 vs. 6.7%, *p* < 0.0001), the medical necessity for tracheotomy (28.6 vs. 1.0%, *p* < 0.0001) as well as the rate of secondary re-intubation (25.6 vs. 4.8%, *p* = 0.0013) were more frequent for patients with CI (Suppl. Table 2). The rate of postoperative bleeding was also elevated in CI group (14.3 vs. 3.8%, *p* = 0.0446). The patients with CI received transfusions of RBCs more often and in higher volumes (frequency, 42.9 vs. 19.2%; *p* = 0.0074; volume, 3.2 ± 1.0 vs. 0.7 ± 0.2 units; *p* = 0.0001) than patients in the control group. The same was true for FFP transfusions (frequency, 31.4 vs. 10.6%; *p* = 0.0064; volume, 1.5 ± 0.4 vs. 0.4 ± 0.1 units; *p* = 0.0007) during the first three postoperative days. More patients in the CI than in the control group needed postoperative vasopressor therapy (71.4 vs. 28.8%, *p* < 0.0001), and more patients with CI needed vasopressor therapy longer than 24 h (40 vs. 10.6%, *p* < 0.0002). Moreover, the mean duration of vasopressor therapy was nearly 5-fold longer in the CI group (137.3 ± 33.9 vs. 27.7 ± 4.6 h, *p* = 0.0003, Suppl. Table 2).

According to the AKIN criteria, the overall incidence of postoperative acute kidney injury was higher in the CI group compared with patients without CI (77.1 vs. 53.8%, *p* = 0.0171, Suppl. Table 3). Moreover, the severity of kidney injury in CI patients was also higher compared with the control group. Most non-CI patients with kidney injury were classified as AKIN I (62.5%), while CI patients mostly developed AKIN II (63.0%) or III (25.9%) renal dysfunction. Moreover, the rate of patients who received extracorporeal renal dialysis in the postoperative period was more than fivefold higher in the CI group (34.3 vs. 5.8%, *p* < 0.0001).

Compared with the control group, both the ICU stay (18.4 ± 2.1 vs. 2.4 ± 0.4 days, *p* < 0.0001) and hospitalisation (48.1 ± 8.5 vs. 14.8 ± 0.7 days, *p* < 0.0001) were significantly longer in the CI group. Eleven patients died during the in-hospital treatment in the CI group and three in the control group. Thus, in-hospital mortality in CI patients was higher than in the control group (31.4 vs. 2.9%, *p* = 0.0001; odds ratio, 15.43; 95% confidence interval, 3.9–59.6).

**Fig. 1:**
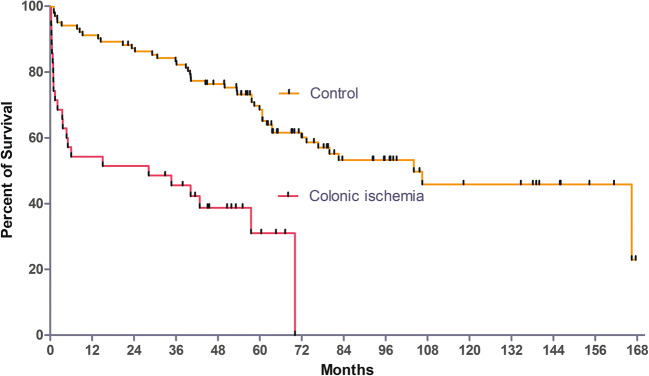
Survival in patients with/without colonic ischaemia after aortic surgery. Survival of the patients with CI was significantly worse compared to the control group (log-rank test: *p* < 0.0001)

**Fig. 2:**
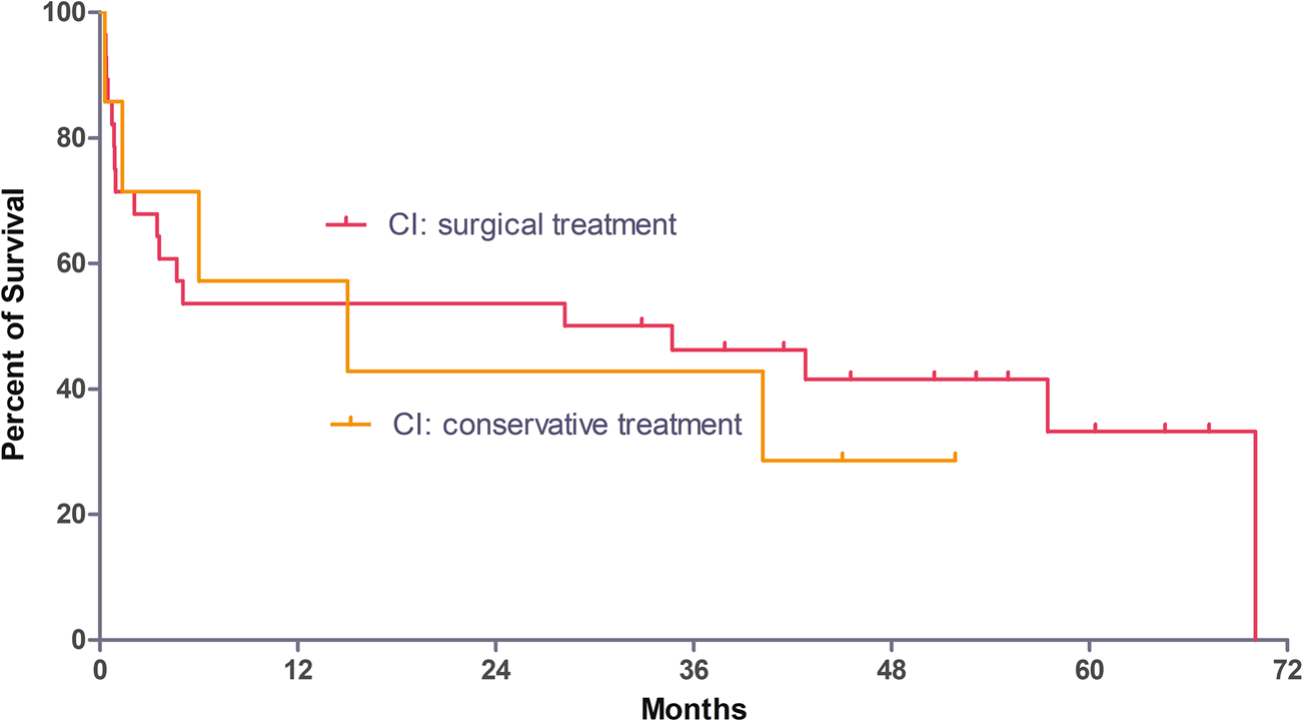
Survival in patients with colonic ischaemia after aortic surgery undergoing surgical/conservative treatment. There was no statistical difference in survival between patients undergoing surgical/conservative treatment (log-rank test: *p* = 0.8822)

FU was available for all 35 patients with CI as well as for 102 patients of the control group. Five patients in the CI group and 64 patients in the control group were still alive after a median FU of 57.5 months (range, 0–168 months; first quartile, 30.6 months; third quartile, 79.2 months). Kaplan–Meier survival curves of patients with and without CI are shown in Fig. 1. Survival of patients with CI (median 28.2 months; first quartile, 1.2 months; third quartile, 48.1 months) was significantly worse (*p* < 0.0001) compared with the control group (median 104.1 months; first quartile, 45.4 months; third quartile, 90.1 months). The estimated 1-, 2-, 3- and 5-year survival rates were 54, 51, 42 and 11% for the CI group and 91, 86, 82 and 59% for the control group. There was no difference in long-term FU between patients with CI who needed surgical treatment and those who did not (Fig. 2).

## Discussion

In the current study, CI after aortic surgery was observed in 2.4% of patients and was associated with a 15-fold–increased in-hospital mortality as well as with significantly reduced survival during FU (Fig. 1).

Patients suffering from rAAA are especially prone to CI [7] due to perioperative blood loss, need for transfusion, retroperitoneal hematoma and abdominal compartment syndrome [3, 8]. Herein, aneurysm rupture considerably increased the prevalence of CI. Therefore, diagnosis of CI after aortic surgery requires a high index of suspicion, especially in patients with rAAA. Current literature suggests that EVAR is associated with lower rates of CI compared to OR [4, 5]. For example, Williamson and colleagues reported in their meta-analysis on 162.750 elective AAA patients (78.151 EVAR and 84.599 OR) and found a combined odds ratio of 2.7 (confidence interval, 2.0–3.5) for the development of CI with OR versus EVAR. Interestingly, this analysis did not reveal any differences between EVAR and OR with respect to CI occurrence, most probably due to the limited number of subjects available for analysis.

In this study, CI was diagnosed in 20 of 35 within the first four postoperative days. This observation is similar to data from 1974 [9] and underlines the need for a high level of vigilance towards this complication within this critical time frame. Nevertheless, in 10 patients CI was detected after the 10^th^ postoperative day. In such delayed cases, which can occur even after 30 days from surgery [4, 8], CI may be associated with a different pathological process. For example, it is possible that CI is not related to surgery (alone), but a result of a prolonged postoperative term in the development of non-occlusive mesenteric hypoperfusion syndrome [10]. If such patients are still under lung ventilation and cannot express the cardinal abdominal symptoms, colonoscopy is helpful in the case of unclear worsening of the general condition. Therefore, the treating physicians must have a high index of suspicion in case of general deterioration and initiate colonoscopy in order to rule out CI. In this study, about half of cases showed general signs of deterioration (i.e. oliguria, circulatory instability). Remarkably, elevated serum lactate occurred for only 31% of patients with CI, emphasising the low sensitivity of this laboratory test. Elevated lactate indicates non-specific tissue ischaemia, mostly as a sign of shock [11]. Thus, the absence of elevated serum lactate should not delay the diagnostic work-up for CI.

Endoscopy plays a major role in the diagnosis of CI after aortic surgery [12]. In 74% of patients, CI was detected by endoscopic means and about one fifth (18.4%) could be treated conservatively due to the endoscopic absence of transmural ischaemia. Nevertheless, when only considering patients with CI, there was no difference in survival between those requiring surgical treatment and those who did not (Fig. 2). This finding is novel and underlines the severity of CI, even in the case of ‘uncomplicated’ mucosal ischaemia. In all 28 patients treated by surgical means, surgery for CI concluded with creation of a stoma, and only eight patients (25.8%) received stoma closure during FU. Despite there was no detailed assessment of quality of life during FU, one may assume that this negatively impacts quality of life.

Comparing co-morbidities of patients with and without CI, we found a higher prevalence of cardiovascular and respiratory impairments in patients with CI. Moreover, patients with CI had worse renal function preoperatively compared with the control group. This finding is in line with a description of idiopathic ischaemic colitis [13, 14] as well as with findings in postoperative CI after aortic surgery [15]. Analysis of intraoperative parameters revealed that longer operation time, blood loss, blood transfusion, intraoperative hypothermia and hypotension were risk factors of CI. These data are not surprising because these parameters often indicate aneurysm rupture. In a previous analysis, longer surgery time and blood loss of > 1 litre were predictive factors of CI [16]. Clearly, many of the reported risk factors are unreducible. Therefore, with respect to the herein analysed CI cohort, early routine postoperative colonoscopy (within 48 h after surgery) may be appropriate in patients with high comorbidity profile, complex intraoperative term and/or rAAA.

The preoperative evaluation of colon blood supply is crucial during the planning of aortic surgery. Nevertheless, there is not much evidence regarding the need for preservation of hypogastric or inferior mesenteric arteries [17]. According to a Canadian aneurysm study, the risk of CI increased eightfold when both internal iliac arteries were occluded compared to when at least one of the internal iliac arteries was preserved [18]. In the current guidelines of the Society for Vascular Surgery, the preservation of blood flow to at least one hypogastric artery in the course of open surgery is recommended [19]. Nevertheless, neither the re-implantation of the inferior mesenteric artery [20] nor the preservation of pelvic perfusion with iliac branch devices [2] decreased ischaemic colitis. At the same time, the presence of atherosclerosis was postulated to be complicated for the pelvic circulation in case of interruption of hypogastric arteries [21], probably due to the loss of collateral blood supply through the hypogastric vessels brunches. This eventuality can explain the findings of our study: we detected that the postoperative lesions—either occlusion or relevant stenosis—of both hypogastric arteries were more usual for CI patients, even though we did not find differences in the preoperative colon blood supply in these patients with regard to the patency of inferior mesenteric or hypogastric arteries.

The present study has several limitations. This is a retrospective observational study covering a study period of more than 10 years, which limits work-up of specific aspects in diagnosis, treatment and FU and makes it subject to the inherent biases of retrospective analyses (i.e. detection bias). The study population is subject to heterogeneity with respect to aortic pathologies and procedures performed. Furthermore, the number of patients diagnosed with CI is limited, a factor that reduces the informative value of the statistical analysis performed.

## Conclusion

The herein reported results demonstrate that CI development is multifactorial, including patient- and procedure-related factors. Patients with rAAA, severe cardiovascular morbidity, complicated intraoperative course and/or perioperative deterioration of the hypogastric perfusion are especially prone to suffer CI. CI is associated with drastic in-hospital mortality and negatively affects survival after discharge, even in patients treated in a conservative fashion. The rate of stoma closure over time is low, a factor that may impact quality of life. Vigilant postoperative multimodal monitoring is required to suspect, detect and, if necessary, treat CI at the earliest.

## Electronic supplementary material

ESM 1 (DOCX 18 kb)
