# Supplementary material for: Outcome analysis and risk factors for postoperative colonic ischaemia after aortic surgery
Source: Langenbecks Arch Surg. 2020 Aug 21;405(7):1031–8. doi: 10.1007/s00423-020-01964-2 (PMC7541358; doi:10.1007/s00423-020-01964-2)
Supplement: Supplementary file 1 — (DOCX 18 kb) [file 423_2020_1964_MOESM1_ESM.docx]

Suppl. Table 1. Intraoperative parameters of matched patients with/without colonic ischaemia

|  | **CI (n=35)** | **Control (n=104)** | **P** | **Odds Ratio** |
| --- | --- | --- | --- | --- |
| Operation time (min) | 283 ± 22 | 188 ± 7 | **<0.0001** |  |
| Blood loss (ml) | 2174 ±396 | 1319 ± 108 | **0.0049** |  |
|  |  |  |  |  |
| Blood loss ≥2000 ml | 15 (42.9%) | 16 (15.4%) | **0.0017** | **2.79 (1.54 - 5.03)** |
| Transfusion of red blood cells | 20 (57.1%) | 32 (30.8%) | **0.0082** | **3,00 (1.36 - 6.60)** |
| Transfusion of red blood cells (units) | 4.7 ± 1.3 | 1.6 ± 0.4 | **0.0021** |  |
| Transfusion of fresh frozen plasma | 20 (57.1%) | 32 (30.8%) | **0.0082** | **3.0 (1.36 - 6.60)** |
| Transfusion of fresh frozen plasma (units) | 4.0 ± 0.9 | 1.4 ± 0.3 | **0.0011** |  |
| Intraoperative hypothermia (< 36°C) | 21 (60%) | 34(32.7%) | **0.0053** | **3.09 (1.40 - 6.81)** |
| Intraoperative hypotension (RRsyst < 90 mmHg) | 14 (40%) | 21 (20.2%) | **0.0251** | **2.64 (1.15 - 6.04)** |

CI: colonic ischaemia.

Suppl. Table 2. Postoperative morbidity of matched patients with/without colonic ischaemia

|  | **CI (n=35)** | **Control (n=104)** | **P** | **Odds Ratio** |
| --- | --- | --- | --- | --- |
| Mechanical ventilation >48h | 14 (40%) | 7 (6.7%) | **<0.0001** | **9,24 (3.32 - 25.69)** |
| Tracheotomy | 10 (28.6%) | 1 (1.0%) | **<0.0001** | **41.20 (5.04 - 337.1)** |
| Secondary  re-intubation | 9 (25.6%) | 5 (4.8%) | **0.0013** | **6.85 (2.12 - 22.21)** |
| Postoperative bleeding | 5 (14.3%) | 4 (3.8%) | **0.0446** | **4.17 (1.05 - 16.51)** |
|  | | | | |
| Transfusion of red blood cells first 3 po days | 15 (42.9%) | 20 (19.2%) | **0.0074** | **3.15 (1.37 to 7.21)** |
| Transfusion of red blood cells first 3 po days (units) | 3.2 ± 1.0 | 0.7 ± 0.2 | **0.0001** |  |
| Transfusion of fresh frozen plasma first 3 po days | 11 (31.4%) | 11 (10.6%) | **0.0064** | **3,88 (1.50 - 10.01)** |
| Transfusion of fresh frozen plasma first 3 po days (units) | 1.5 ± 0.4 | 0.4 ± 0.1 | **0.0007** |  |
|  | | | | |
| Postop. vasopressors | 25 (71.4%) | 30 (28.8%) | **<0.0001** | **6,17 (2.64 - 14.39)** |
| Postop. vasopressors >24h | 14 (40%) | 11 (10.6%) | **0.0002** | **5,76 (2.29 - 14.46)** |
| Duration vasopressors usage [h] | 137.3 ± 33.9 | 27.7 ± 4.6 | **0.0003** |  |

CI: colonic ischaemia.

Suppl. Table 3. Postoperative acute kidney injury of matched patients with/without colonic ischaemia

|  | **CI (n=35)** | **Control**  **(n=104)** | **P** | **Odds Ratio** |
| --- | --- | --- | --- | --- |
| Acute kidney injury (AKIN) | 27 (77.1%) | 56 (53.8%) | **0.0171** | **2,89 (1.20 - 6.96)** |
| AKIN I  Increase in serum creatinine ≥ 0,3 mg/dl or 150-200% from baseline | 3 (11.1%) | 35 (62.5%) | **<0.0001** | **0.08 (0.02 - 0.28)** |
| AKIN II  Increase in serum creatinine 200% to 300% from baseline | 17 (63.0%) | 16 (28.6%) | **0.0040** | **4.25 (1.61 - 11.24)** |
| AKIN III  Increase in serum creatinine >200% from baseline | 6 (25.9%) | 5 (8.9%) | 0.1633 | 2.91 (0.80 - 10.60) |
|  |  |  |  |  |
| Novel postoperative haemodialysis (all temporary) | 12 (34.3%) | 6 (5.8%) | **<0.0001** | **8.52 (2.89 - 25.10)** |
| Max postoperative level of Creatinine | 2.9 ± 0.2 | 1.9 ± 0.13 | **<0.0001** |  |

CI: colonic ischaemia, AKIN: acute kidney injury network.
